# Development of a Multivariate Prognostic Model for Lenvatinib Treatment in Hepatocellular Carcinoma

**DOI:** 10.1093/oncolo/oyad107

**Published:** 2023-04-27

**Authors:** Xiaomi Li, Xiaoyan Ding, Mei Liu, Jingyan Wang, Wei Li, Jinglong Chen

**Affiliations:** Department of Cancer Center, Beijing Ditan Hospital, Capital Medical University, Beijing, People’s Republic of China; Department of Cancer Center, Beijing Ditan Hospital, Capital Medical University, Beijing, People’s Republic of China; Department of Oncology, Beijing You’an Hospital, Capital Medical University, Beijing, People’s Republic of China; Department of Interventional Radiology, The Fifth Medical Center, Chinese PLA General Hospital, Beijing, People’s Republic of China; Department of Cancer Center, Beijing Ditan Hospital, Capital Medical University, Beijing, People’s Republic of China; Department of Cancer Center, Beijing Ditan Hospital, Capital Medical University, Beijing, People’s Republic of China

**Keywords:** hepatocellular carcinoma, Lenvatinib, prognostic model, prognostic nutritional index, platelet-to-lymphocyte ratio

## Abstract

**Background:**

Lenvatinib is a first-line agent for advanced hepatocellular carcinoma (HCC), but individual responses to treatment are highly heterogeneous. The aim of this study was to investigate the clinical parameters that influence the efficacy of Lenvatinib and to develop a prognostic model.

**Methods:**

We retrospectively enrolled 333 Lenvatinib-treated patients with HCC with a median age of 57 years. Two hundred nd sixty-three of these patients had BCLC (2022) stage C. The median overall survival (mOS) time within the cohort was 12.1 months, and the median progression-free survival (mPFS) time was 4.7 months. Univariate Cox regression, best subset regression, and Lasso regression were used to screen primary variables for possible contribution to OS, multivariate Cox analysis was used to fit selected models, and the final model was selected using the maximum area under the curve (AUC) and minimum AIC. Receiver operating curves (ROC), calibration curves, and decision curve analysis were plotted to assess model performance, and 5-fold cross-validation was performed for internal validation. X-tile software was used to select the best cutoff points and to divide the study cohort into 3 different risk groups.

**Results:**

Seven variables were included in the final model: BCLC stage, prior transarterial chemoembolization and immunotherapy history, tumor number, prognostic nutritional index, log (alpha-fetoprotein), and log (platelet-to-lymphocyte ratio). We named this final model the “multivariate prognostic model for Lenvatinib” (MPML), and a nomogram was constructed to predict the probability of survival at 6, 9, and 12 months. The MPML had good discrimination, calibration, and applicability. Cross-validation showed mean AUC values of 0.7779, 0.7738, and 0.7871 at 6, 9, and 12 months, respectively. According to nomogram points, mOS time was 21.57, 8.70, and 5.37 months in the low, medium, and high-risk groups, respectively (*P* < .001), and these differences were also observed in the PFS survival curve (*P* < .001).

**Conclusions:**

The MPML stratified patients according to baseline clinical characteristics had a strong performance in predicting Lenvatinib efficacy and has the potential for use as an auxiliary clinical tool for individualized decision-making.

## Teaser

Lenvatinib is a first-line agent for advanced hepatocellular carcinoma, but individual responses to treatment are highly heterogeneous. This study investigated the clinical parameters that influence the efficacy of Lenvatinib, to develop a prognostic model.

**Implications for Practice**

The authors found that BCLC stage, prior transarterial chemoembolization, and immunotherapy history, tumor number, prognostic nutritional index, log (alpha-fetoprotein), and log (platelet-to-lymphocyte ratio) have important clinical significance, and constructed nomograms and developed online access tools https://xiaomili.shinyapps.io/MPML/. The model had good discrimination, calibration, and practicability, and divided the patient population into 3 risk groups with significant prognostic differences.

## Introduction

Primary liver cancer is the sixth-most common cancer, and the third leading cause of cancer death worldwide, with hepatocellular carcinoma (HCC) accounting for 75%-85% of cases.^1^ Early HCC can be cured by local therapy, but moderate HCC, which progresses after transarterial chemoembolization (TACE) and advanced HCC both have poor prognoses and require systemic therapy.^2,3^ Sorafenib was the first tyrosine kinase inhibitor (TKI) approved for advanced HCC based on several trials (ie, SHARP and Asia-Pacific), which showed good efficacy.^4,5^ However, Sorafenib’s objective response rate (ORR) was less than 5%, median overall survival (mOS) time was prolonged by less than 3 months, and the drug had some side effects.^4,5^ Another TKI, Lenvatinib has been shown to be non-inferior to Sorafenib in OS and was approved in 2018 for first-line treatment of unresectable HCC.^6^ In addition to targeted agents, the advent of immunotherapy has provided new treatment options for advanced HCC, and target-immunity combinations have synergistic sensitizing effects.^7,8^

Despite major advances in systemic therapy in recent years, TKIs remain the cornerstone drugs for moderate and advanced HCC. Sorafenib was the only treatment option for advanced HCC for many years, and several groups have identified potential prognostic factors for Sorafenib response, including neutrophil-to-lymphocyte ratio (NLR), glasgow prognostic score, lymphocyte-to-monocyte ratio (LMR), and systemic immune-inflammatory (SII) levels.^9-12^ In addition, several prognostic models (ie, SAP and PROSASH) have been developed to individualize survival prediction in Sorafenib-treated HCC.^13-17^ Lenvatinib inhibits vascular endothelial growth factor receptor (VEGFR) 1-3, platelet-derived growth factor receptor (PDGFR) α, fibroblast growth factor receptor (FGFR) 1-4, KIT, and RET, thus inhibiting tumor cell proliferation and exerting anti-angiogenesis effects. Lenvatinib targets are more concentrated and inhibitory than Sorafenib targets, and some studies, including the REFLECT trial, have demonstrated significant improvements in median ­progression-free survival (mPFS), ORR, and time to tumor progression among patients with HCC treated with Lenvatinib.^6^ However, even in the REFLECT trial, the ORR was less than 30%, the disease control rate (DCR) was less than 70%, and the incidence of grade 3 and higher adverse events (AEs) was high, suggesting that appropriate patient selection is critical prior to treatment. Several studies have found that controlling nutritional status scores,^18^ the prognostic nutritional index (PNI),^19^ and muscle volume reduction^20^ all have high-predictive value. Using recursive partitioning analysis, Rapposelli et al constructed a LEP index that included 4 variables (PNI, previous TACE history, ALBI, and BCLC stage) and divided Lenvatinib-treated patients into 3 risk cohorts with significant between-group differences.^21^

Although these clinical and molecular markers have been found to predict treatment responses to Lenvatinib, robust data on prognostic factors remain sparse. We propose to combine different factors and develop a new prognostic model. We hope our model may eventually serve as an adjunctive clinical tool to individualize the prediction of patient outcomes.

## Methods

### Patient Selection

The study population was composed of patients who were diagnosed with HCC and treated with Lenvatinib at 3 centers in Beijing between October 2017 and October 2021. Criteria for enrollment included: (1) age >18 years, (2) ECOG PS 0-2, (3) Child-pugh grade A or B, and (4) diagnosis of HCC confirmed by imaging and histological examination. Patients were also excluded if they (1) had a history of malignant tumors other than HCC, (2) had a significant coagulation disorder or tendency toward active bleeding, (3) were pregnant or lactating, or (4) had contraindications to Lenvatinib. Patients with HCC who had received or were actively receiving immunotherapy were still permitted to enroll in the study.

This retrospective study was in accordance with the clinical practice guidelines and the Declaration of Helsinki 1975 and was approved by the Ethics Committee of Beijing Ditan Hospital, Capital Medical University. Lenvatinib treatment was started after obtaining written informed consent from each patient. Lenvatinib was administered orally at 8 mg/day for patients weighing <60 kg and 12 mg/day for patients weighing ≥60 kg and was discontinued if unacceptable or serious AE or clinical tumor progression was observed. Response to treatment was assessed using modified Response Evaluation Criteria in Solid Tumors (mRECIST) guidelines 4 to 6 weeks after starting Lenvatinib, and every 2 to 3 months thereafter until death or study termination.^22^

### Data Collection

Baseline data were collected before the initiation of Lenvatinib therapy. Disease progression and patient survival were also recorded. Clinical information included age, gender, ECOG PS, etiology, and previous treatment history (hepatectomy, ablation, TACE, and immunotherapy); imaging data included tumor size, tumor number, presence or absence of portal vein tumor thrombus (PVTT), and extrahepatic metastasis (EHM); serological indicators included complete blood count (absolute neutrophil, lymphocyte, monocyte, and platelet counts), liver function (albumin and bilirubin), coagulation function (prothrombin time), and alpha-fetoprotein (AFP). Participants were divided into a hepatitis B virus group, a hepatitis C virus group, and an “other” group depending on tumor etiology. Tumor number (“≤3″ and “>3”) and size (“≤5 cm” and “>5 cm”) were treated as dichotomous variables. Lenvatinib treatment lines were also reviewed and documented. Patients were classified using BCLC staging guidelines,^23^ and the following scores were calculated using the collected data: Child-Pugh score, NLR, platelet-to-lymphocyte ratio (PLR), LMR, SII, and PNI. The primary outcome measure in this study was OS, defined from the initiation of Lenvatinib treatment until death from any cause or the date of the last follow-up. PFS was defined as the time from the initiation of Lenvatinib treatment until disease progression or death from any cause.

### Statistical Analysis

Statistical analyses and graphs were completed and compiled using R software (version 4.1.1). Normally distributed continuous variables are presented as means ± (standard deviations), non-normally distributed continuous variables are presented as medians ± interquartile ranges, and categorical variables are presented as counts (percentages). Histograms were used to determine variable distributions, and logarithmic transformation was performed for continuous variables with a clearly skewed distribution. Survival curves were plotted using the Kaplan-Meier method, and median survival and 95% confidence intervals (95% CI) were reported.

### Model Build and Validation

To select the best model, we first screened variables using univariate Cox regression, best subset regression (BSR), and Lasso regression. We next used multivariate Cox backward stepwise regression to fit each of these 3 initial models. Finally, we drew receiver operating curves (ROCs) and calculated areas under the curve (AUCs) for each model and selected the model with the largest AUC as our final prediction model. Using the selected variables, nomogram and web page were constructed to predict the probability of survival at 6, 9, and 12 months. The final model was also assessed by plotting ROC, calibration curve, and decision curve analysis (DCA) using OS at one year as the time node. We used cross-validation for internal validation, with results presented using box and scatter plots. All patients were scored according to the model, and the best cutoff value was selected using X-tile software. Our final model divided patients into 3 groups: a high-risk group, a medium-risk group, and a low-risk group. Finally, between-group survival differences in OS and PFS were visually compared to demonstrate the effectiveness of the model.

## Results

### Baseline Patient Characteristics

A total of 351 patients with HCC were treated with Lenvatinib at our study sites between October 2017 and October 2021. After the exclusion of 18 ineligible patients, the remaining 333 patients constituted the study cohort (Supplementary Fig. S1). The median age was 57 (range 50-64) years, and 84.4% of participants were male (*n* = 281). All included patients had moderate or advanced stage disease, with the majority having BCLC stage C disease (*n* = 263, 79.0%). The remaining baseline characteristics are summarized in Table 1.

**Table 1.** Patient baseline characteristics.

Characteristic	Value (*n* = 333)
Age, median [range]	57.0 [50.0, 64.0]
NLR, median [range]	3.0 [2.0, 4.4]
PLR, median [range]	117.7 [80.0, 171.4]
LMR, median [range]	2.5 [1.7, 3.6]
PNI, median [range]	42.3 [37.3, 46.9]
SII, median [range]	379.3 [220.5, 648.2]
AFP, median [range]	88.7 [6.5, 2000.0]
Sex, *n*(%)	
Male	281 (84.4)
Female	52 (15.6)
Etiology, *n*(%)	
HBV	291 (87.4)
HCV	31 (9.3)
Others	11 (3.3)
ECOG PS, *n*(%)	
0	163 (48.9)
1	143 (42.9)
2	27 (8.1)
Number, *n*(%)	
≤3	133 (39.9)
>3	200 (60.1)
Size, *n*(%)	
≤5 cm	206 (61.9)
>5 cm	127 (38.1)
PVTT, *n* (%)	
0	153 (45.9)
I	28 (8.4)
II	69 (20.7)
III	54 (16.2)
IV	29 (8.7)
Metastasis, *n*(%)	134 (40.2)
Prior therapy, *n*(%)	
Recection	51 (15.3)
Ablation	145 (43.5)
TACE	286 (85.9)
ICI	187 (56.2)
Lenvatinib treatment line	
First line	251 (75.4)
Second line	77 (23.1)
Third line	5 (1.5)
Child-Pugh score, *n* (%)	
5	121 (36.3)
6	95 (28.5)
7	60 (18.0)
8	31 (9.3)
9	26 (7.8)
BCLC stage, *n* (%)	
B	70 (21.0)
C	263 (79.0)

Abbreviations: NLR, neutrophil-to-lymphocyte ratio; PLR, ­platelet-to-lymphocyte ratio; LMR, lymphocyte-to-monocyte ratio; PNI, prognostic nutritional index; SII, systemic immune-inflammatory; AFP, alpha-fetoprotein; HBV, hepatitis B virus; HCV, hepatitis C virus; ECOG PS, Eastern Cooperative Oncology Group performance status; PVTT, portal vein tumor thrombosis; TACE, transarterial chemoembolization; ICI, immune checkpoint inhibitor.

### Response to Treatment

At the last follow-up time point, 169 participants had died, and 238 participants had experienced disease progression. Survival analysis plots for OS and PFS for Lenvatinib-treated patients with HCC are shown in Supplementary Fig. S2, with a mOS of 12.1 (95% CI, 10.4-15.4) months and a mPFS of 4.7 (95% CI, 3.97-5.73) months. The OS rates at 6, 9, and 12 months were 60.1%, 41.7%, and 29.4%, respectively. Treatment responses were assessed according to mRECIST criteria in 296 patients, with 3 complete responses, 33 partial responses, 142 patients with stable disease, and 118 patients with progressive disease, resulting in an ORR of 12.2% and a DCR of 60.1%.

### Variable Screening

Variable screening is a critical step in model development. We exponentially transformed NLR, PLR, LMR, SII, and AFP values because they had skewed distributions. Next, we used Cox regression to assess OS, with potential predicting variables including the history of ablation, history of TACE, history of immunotherapy, BCLC stage, number of tumors, ECOG PS, Child-Pugh score, PVTT, EHM, PNI, and NLR, PLR, LMR, SII, and AFP indices (Table 2). We included these variables in a multivariate Cox regression analysis, with the final fitted model termed “COX” (BCLC + TACE + ICI (immune checkpoint inhibitor) + Number + PNI + log [AFP] + log [PLR]). Next, we developed a model using the BSR approach, with variables including sex, ablation history, and immunotherapy history, BCLC stage, tumor number, PNI, and LMR and AFP indices (Supplementary Fig. S3). This fitted model was also judged using the stepwise backward method and was termed “BSR” (BCLC + Albation + ICI + Number + PNI + log [AFP]). Finally, we generated a model using Lasso regression, with the initial variables including ablation history, TACE history, immunotherapy history, BCLC stage, number of tumor tumors, ECOG PS, Child-Pugh score, PVTT, EHM, and PNI (Supplementary Fig. S4). We used multivariate Cox regression to select the model with the smallest AIC and termed it “LASSO” (BCLC + TACE + ICI + Number + Metastasis + PNI). ROC curves were plotted for the 3 models, and each model’s AUC was calculated as shown in Supplementary Fig. S5. The “COX” model had the largest AUC value and the smallest AIC value and was, therefore, selected as the final model.

**Table 2.** Univariate Cox regression for OS.

Characteristic	HR (95% CI)	*P*
Sex (male vs. female)	1.14 (0.73-1.78)	.575
Etiology (HCV vs. HBV)	0.71 (0.42-1.2)	.202
Etiology (other vs. HBV)	1.25 (0.51-3.06)	.621
Number (>3 vs. ≤3)	1.67 (1.21-2.31)	.002
Size (>5 cm vs. ≤5 cm)	0.9 (0.65-1.23)	.501
Resection (yes vs. no)	0.66 (0.42-1.03)	.064
Ablation (yes vs. no)	0.7 (0.52-0.95)	.023
TACE (yes vs. no)	0.4 (0.26-0.63)	0
ICI (yes vs. no)	0.59 (0.44-0.8)	.001
Lenvatinib treatment line (second vs. first)	1.08 (0.77-1.51)	.663
Lenvatinib treatment line (third vs. first)	0.92 (0.34-2.5)	.87
Metastasis (yes vs. no)	1.75 (1.29-2.37)	0
BCLC stage (C vs. B)	2.39 (1.54-3.72)	0
Age	1 (0.98-1.01)	.938
ECOG PS	1.35 (1.06-1.71)	.016
Child-Pugh score	1.33 (1.18-1.49)	0
PVTT	1.2 (1.08-1.33)	.001
PNI	0.95 (0.92-0.97)	0
log(NLR)	2.83 (1.63-4.92)	0
log(PLR)	3.55 (1.83-6.86)	0
log(LMR)	0.45 (0.26-0.79)	.006
log(SII)	1.93 (1.27-2.94)	.002
log(AFP)	1.23 (1.1-1.38)	0

Abbreviations: OS, overall survival; HR (95%CI), hazard ratio (95% confidence interval); HBV, hepatitis B virus; HCV, hepatitis C virus; TACE, transarterial chemoembolization; ICI, immune checkpoint inhibitor; ECOG PS, Eastern Cooperative Oncology Group performance status; PVTT, portal vein tumor thrombosis; PNI, prognostic nutritional index; NLR, neutrophil-to-lymphocyte ratio; PLR, platelet-to-lymphocyte ratio; LMR, lymphocyte-to-monocyte ratio; SII, systemic immune-inflammatory; AFP, alpha-fetoprotein.

### Model Development

**Figure 1.**
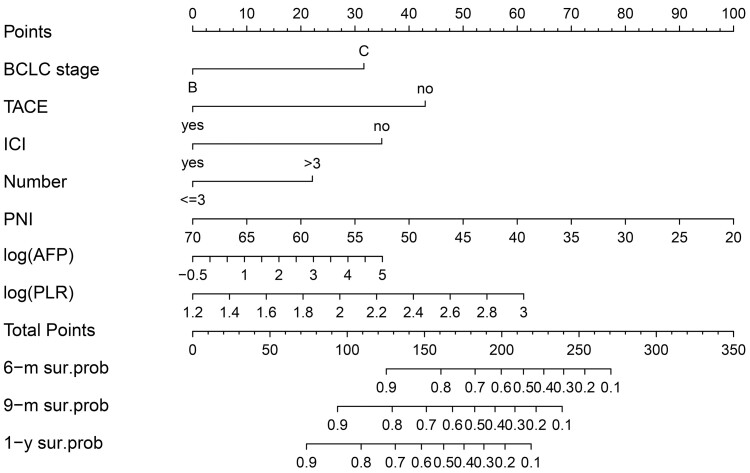
Nomogram for the multivariate prognostic model for Lenvatinib (MPML).

We generated a nomogram to predict the probability of survival at 6, 9, and 12 months based on 7 variables: BCLC stage, previous TACE and immunotherapy history, tumor number, PNI, log (AFP), and log (PLR) (Fig. 1). This final model was termed the “multivariate prognostic model for Lenvatinib” (MPML). We also generated an online tool using the MPML to facilitate application for individualized clinical prediction: https://xiaomili.shinyapps.io/MPML/.

### Model Evaluation and Validation

**Figure 2.**
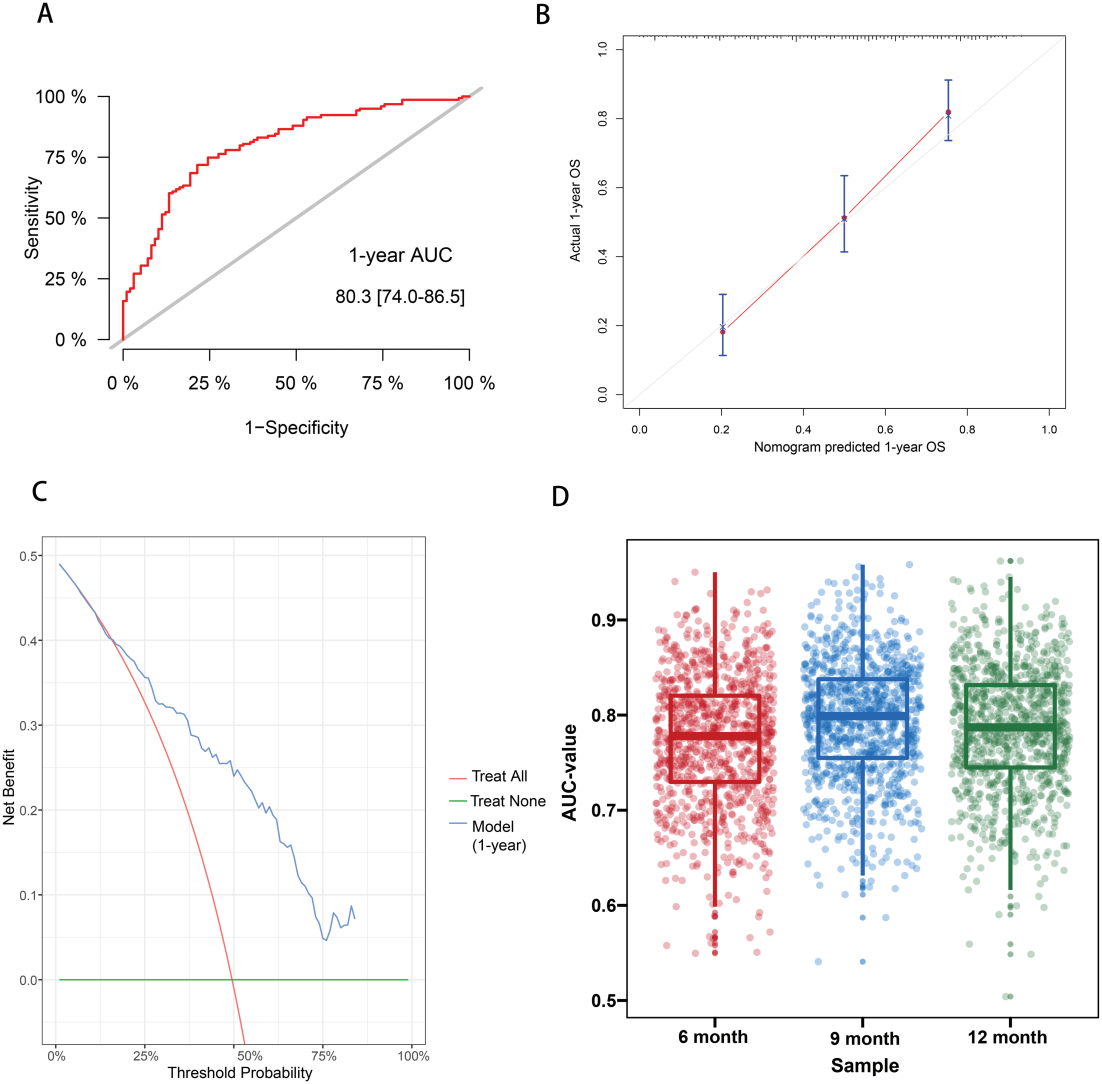
Receiver operating curve (**A**), calibration curve (**B**), and decision curve analysis (**C**) for model prediction of one-year survival, and Box and scatter plots of the area under the internal validation curve for 6, 9, and 12 months (**D**).

To evaluate the MPML’s performance, we plotted ROC, calibration curve, and DCA, with one year as the time node. The AUC value predicted by the one-year survival probability of this model was 0.803 (range 0.74-0.865), which had good discriminatory power (Fig. 2A). In addition, the nomogram predicted a survival probability at one year that was not substantially different from actual patient outcomes, suggesting good consistency (Fig. 2B). Finally, DCA assessed the utility of the model and found an evident positive net benefit predicted by the one-year survival probability (Fig. 2C). Fig. 2D presents the internal cross-validation results, with mean AUC values of 0.7779 (range 0.7298-0.7738), 0.7738 (range 0.7550-0.7942), and 0.7871 (range 0.7449-0.7860) at the 6, 9, and 12-month timepoints, respectively.

### Risk Stratification

**Figure 3.**
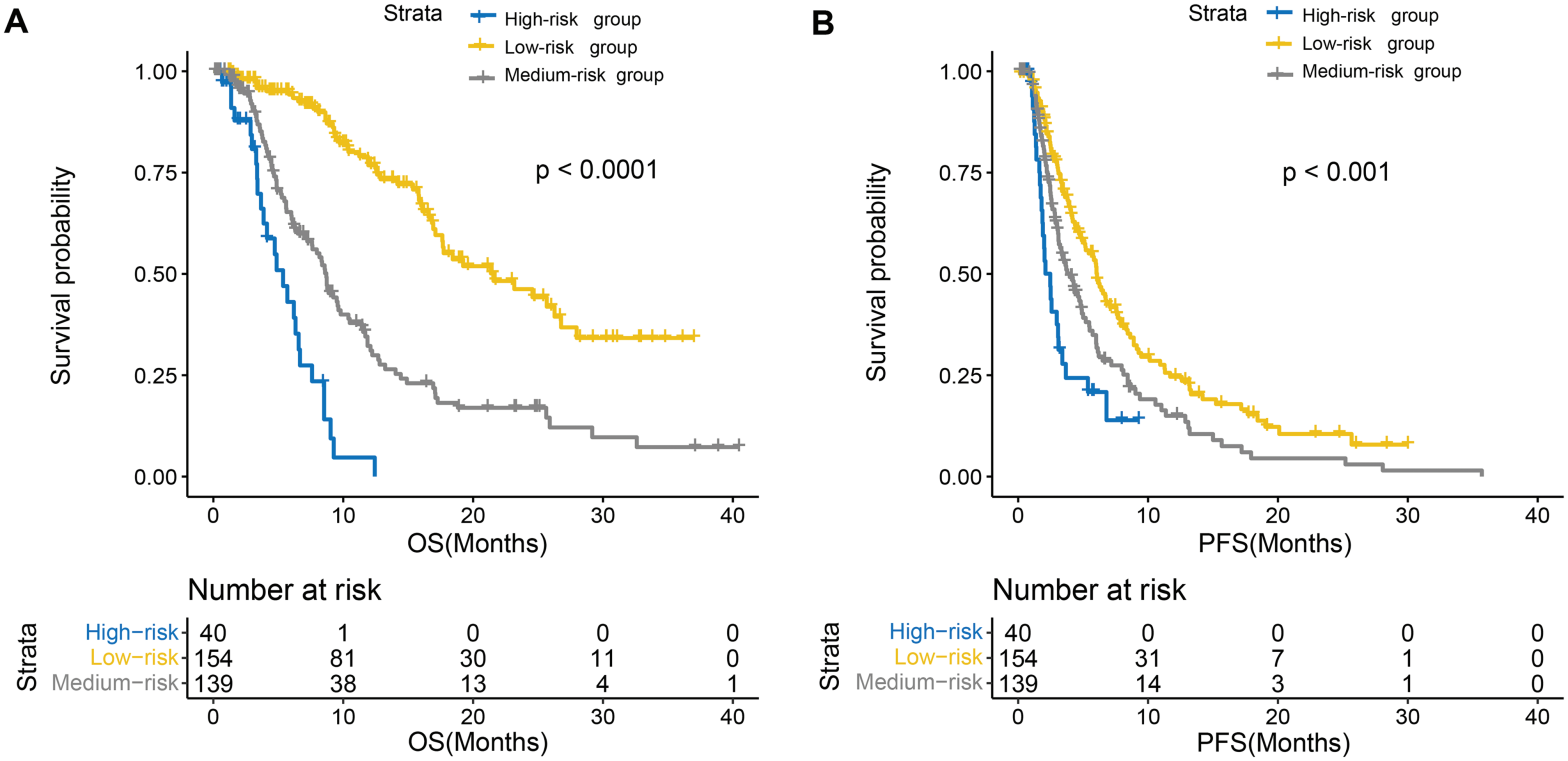
Kaplan-Meier curves for overall survival (**A**) and progression-free survival (**B**) across risk groups.

Our study population was scored individually according to the score shown on the nomogram, and patients were divided into 3 risk groups—high, medium, and low—using X-tile software with best cutoff values of 157.6 and 206. Of these, 154 patients were included in the low-risk group, with a longer mOS (21.57 months; 95% CI, 17.63-28.0 months) and mPFS (6.07 months; 95% CI, 5.10-7.6 months); the medium-risk group had 139 patients, with a mOS of 8.70 (95% CI, 7.23-10.4) months and a mPFS of 3.97 (95% CI, 3.13-5.0) months; and the remaining 40 patients were in the high-risk group, with a shorter mOS (5.37 months; 95% CI, 3.87-7.6 months) and mPFS (2.47 months; 95% CI, 1.87-3.4 months). The survival curves are shown in Fig. 3, where we observed clear survival differences in OS and PFS for the different risk groups.

## Discussion

Based on the results of the REFLECT trial, Lenvatinib is now a first-line treatment for advanced HCC, and multiple studies have demonstrated its efficacy and safety. Although Lenvatinib has had widespread clinical application in recent years, some patients do not respond to the drug or experience adverse side effects. Thus, prognostic factors which may predict which subsets of patients with HCC will benefit from Lenvatinib still need to be explored. Here, we screened 7 variables with important clinical significance and drew nomograms to construct a model (termed the “MPML”) using a study cohort of patients with HCC who were treated with Lenvatinib. Using the MPML, patients were divided into 3 risk groups with significant prognostic differences, with an mOS of only 5.37 months in the high-risk group, an mOS of 8.7 months in the medium-risk group, and an mOS of 21.57 months in the low-risk group, and good calibration performance and application ability. In addition, we performed internal validation to assess the model’s stability, which showed it had strong discriminatory abilities at 6, 9, and 12 months.

There are currently no feasible clinical markers that can be used for individualized prediction of Lenvatinib responses. However, our constructed model, which includes tumor and inflammatory markers in addition to tumor characteristics and treatment history, was able to stratify patients with HCC who were treated with Lenvatinib. BCLC staging is one of the most commonly used staging guidelines in HCC clinical practice. Our study cohort was limited to patients with BCLC stages C or B disease who had already failed TACE treatment, in which TKIs, including Lenvatinib, are an essential component of treatment.^23^ The main difference between stages B and C disease is the presence or absence of vascular invasion as well as EHM. Invasive HCC is associated with worse prognoses than nodular HCC, with more pronounced symptoms and/or higher AFP levels.^24^ PVTT and EHM are among the most robust factors which can predict patient death, and metastasis can involve different sites and is an important clinical manifestation of advanced HCC.^10,14-17,25,26^ In this study, we observed an independent predictive effect of advanced HCC on Lenvatinib treatment. Although BCLC is the preferred staging system, it does not include treatment history or tumor characteristics. TACE can be considered for HCC treatment in the moderate disease stage, but it may also lead to local hypoxia and thus stimulate tumor vessel growth.^27^ Several clinical studies have found that a combination of TACE and Lenvatinib is more effective than Lenvatinib alone or TACE-Sorafenib combination therapy.^28-30^ Patients with a history of TACE also had a reduced risk of death in our study, and this history may have impacted their subsequent Lenvatinib treatment outcomes. In addition to targeted agents, immunotherapy is a good option for advanced HCC.^31,32^ TKIs have immunomodulatory effects and may combine anti-angiogenesis with ICI properties.^33^ In addition, Atezolizumab-Bevacizumab combination therapy and Lenvatinib-Pembrolizumab combination therapy have shown promise in clinical trials.^7,8^ Given the clinical significance of prior TACE and immunotherapy history, it is practical to include them in nomograms to predict survival.^11^ As an important variable in the BCLC staging system, tumor number has also been shown to be an independent predictor of HCC survival.^10^ The NIACE score predicted HCC treated with Sorafenib and included this indicator and also suggested that ≥3 tumor nodules were a prognostic factor for survival.^34^

Circulating inflammatory factors and tumor markers are closely associated with HCC development and prognosis. NLR, PLR, PNI, and SII are all immune-inflammatory markers that are based on peripheral blood cell counts.^9,11,35^ PNI is a nutritional prognostic indicator that was calculated using serum albumin and peripheral blood lymphocytes and was developed in a study of postoperative complications associated with gastrointestinal resection.^36^ A meta-analysis involving 3165 patients found that PNI was independently associated with HCC death.^37^ Its predictive value has been demonstrated in 2 prior studies on Lenvatinib treatment.^21,23^ Another effective factor for predicting HCC death is AFP, which is a common tumor marker.^10,11,13-15,25^ In a real-world study of Lenvatinib treatment in Japan, AFP ≥400 ng/mL was an independent pretreatment factor for patient mortality.^38^ PLR is one of the systemic inflammatory factors calculated from platelet and lymphocyte numbers and is associated with poor outcomes in patients with HCC. A ­meta-analysis of 6318 patients showed that high PLR levels before treatment predicted worse OS and relapse-free survival, and the same results were found in the treatment subgroups who received hepatectomies, liver transplantations, ablations, and TACE.^39^ However, there are few relevant studies on Lenvatinib and PLR. Tada et al investigated the predictive ability of low (<150) vs. high (≥150) PLR for Lenvatinib-related outcomes in patients with HCC using inverse probability weighted analysis.^40^ Kariyama et al proposed the simplified albumin (ALBS) classification, which uses albumin (an available biomarker) alone to stratify Lenvatinib-treated patients with HCC and has good utility (c-index 0.765).^25,41^ Variables included in the above scores were associated with several mechanisms, including ­neutrophil-mediated inhibition, anti-tumor immune surveillance, and the promotion of tumor cell invasion, all of which can affect tumor growth and progression in the tumor microenvironment.^42^ Lymphocytes not only reflect nutritional status but are also markers of systemic inflammation. High levels of lymphocytes have antitumor activity, infiltrate into tumor tissue, and induce tumor cell apoptosis.^43^ Tumor cells interact with platelets and promote neutrophil accumulation in tumor tissue, which helps form metastases.^44,45^ In our study, PNI, AFP, and NLR were included as continuous rather than discrete variables, meaning that the MPML incorporates more detailed information than previous models.

Blood counts and liver function tests are routine tests for clinical patients, and PNI, AFP, and NLR are also conveniently obtained as prognostic markers. Other parameters were obtained by consulting imaging data and reviewing case data. Our model contains conventional clinical indicators (ie, AFP and BCLC stage), inflammatory and nutritional indices, and treatment history, making it far more comprehensive.

However, our study also has some limitations. As a retrospective study, we could not control for patients receiving other systemic therapies before or after their Lenvatinib treatments. Our study also had a relatively short-follow-up time period, and prolonged observation is needed to strengthen our model’s credibility. Finally, our model lacks external validation by other institutions, and although we achieved strong internal validation results within our center, further validation is needed.

## Conclusion

We developed a simple and comprehensive prognostic model that combined clinical factors and inflammatory markers to predict HCC survival following Lenvatinib treatment. We encourage other centers to validate this MPML and test its predictive usefulness.

## Supplementary Material

oyad107_suppl_Supplementary_Material

## Data Availability

All data generated or analyzed during this study are included in this published article.
