# Supplementary material for: Development of a Multivariate Prognostic Model for Lenvatinib Treatment in Hepatocellular Carcinoma
Source: Oncologist. 2023 Apr 27;28(10):e942–9. doi: 10.1093/oncolo/oyad107 (PMC10546830; doi:10.1093/oncolo/oyad107)

**Supplemental Data:**

**Supplemental Figure S1:** Study flowchart.

**Supplemental Figure S2:** Kaplan-Meier curves of overall survival (A) and progression-free survival (B) in the whole population.

**Supplemental Figure S3:** Best subset regression results.

**Supplemental Figure S4:** Lasso regression results.

**Supplemental Figure S5:** Receiver operating curves for the three models: "COX", "BSR" and "Lasso".

**Supplemental Figure S1:** Study flowchart.


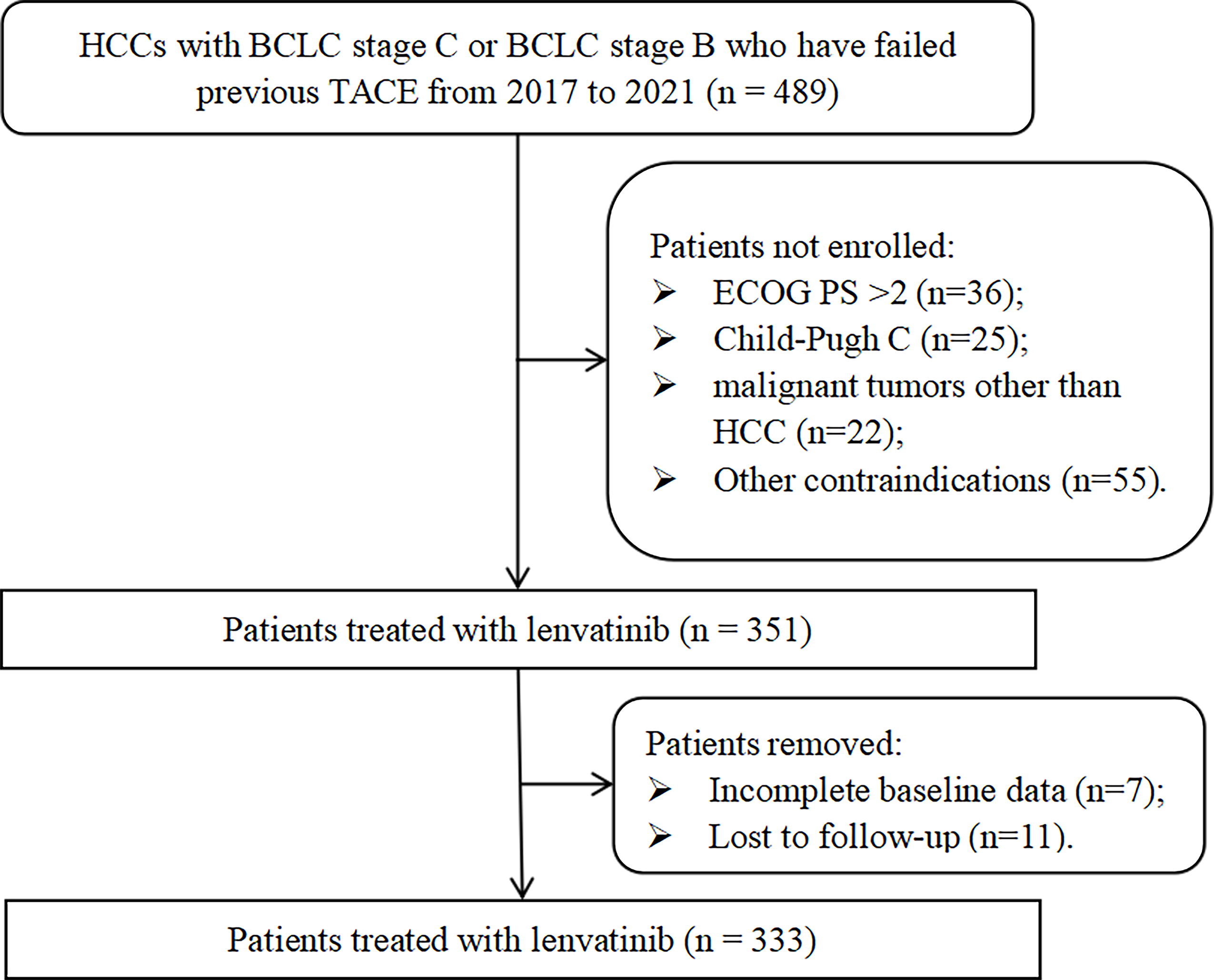


**Supplemental Figure S2:** Kaplan-Meier curves of overall survival (A) and progression-free survival (B) in the whole population.


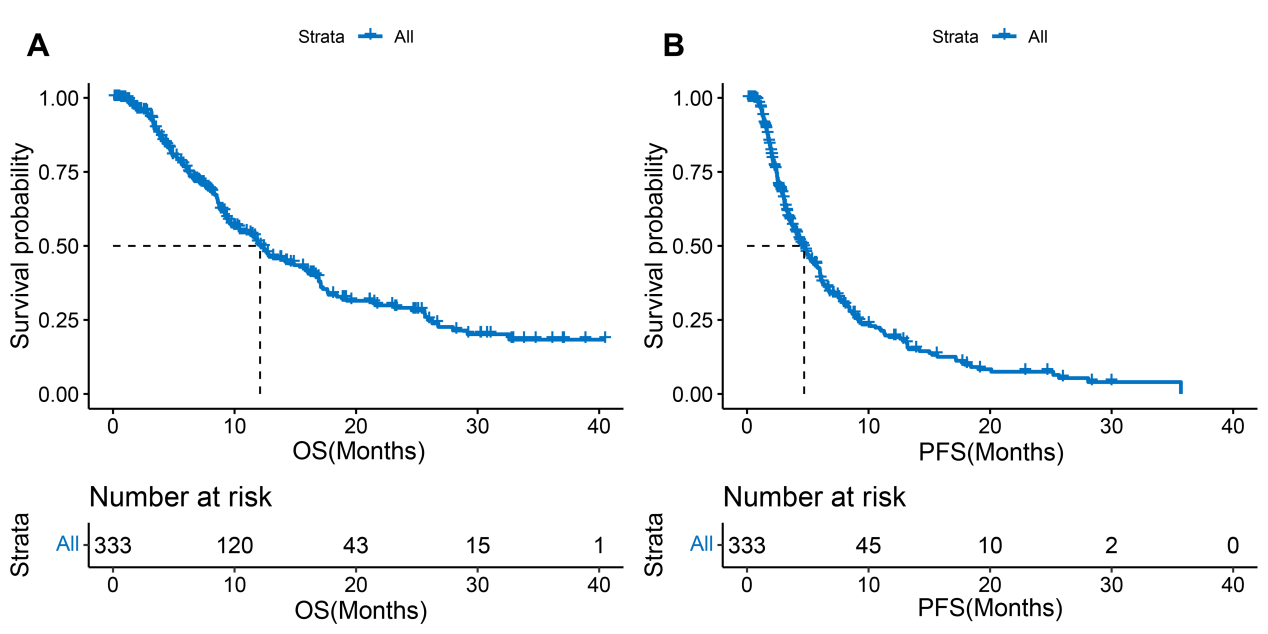


**Supplemental Figure S3:** Best subset regression results.


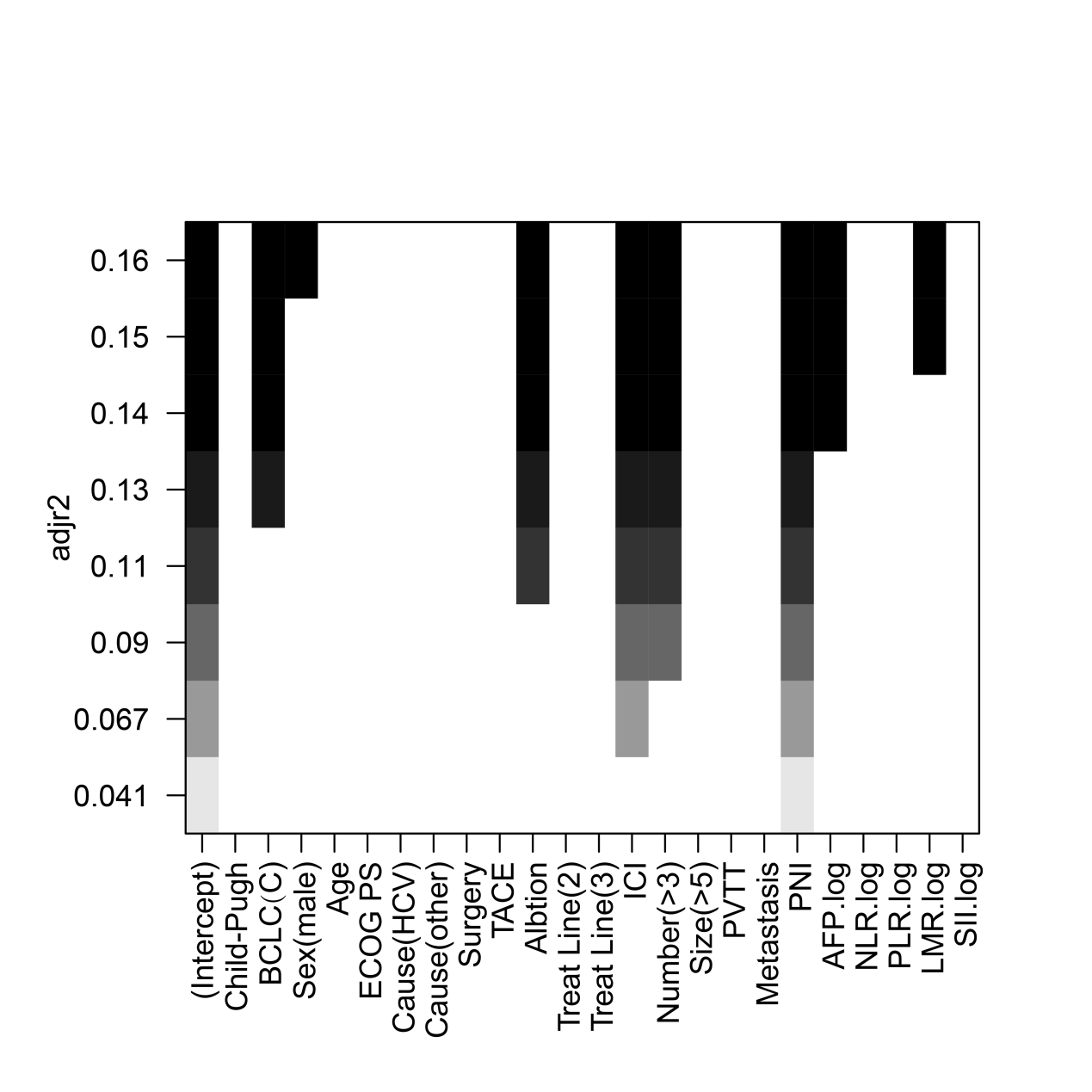


**Supplemental Figure S4:** Lasso regression results.


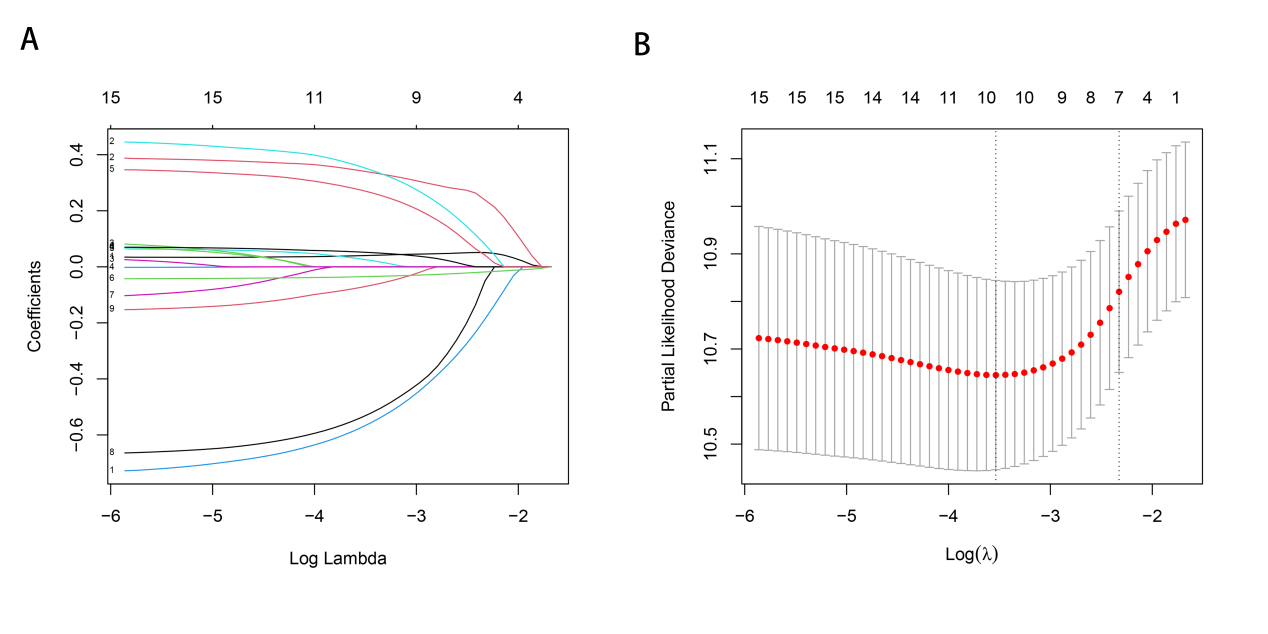


**Supplemental Figure S5:** Receiver operating curves for the three models: "COX", "BSR" and "Lasso".


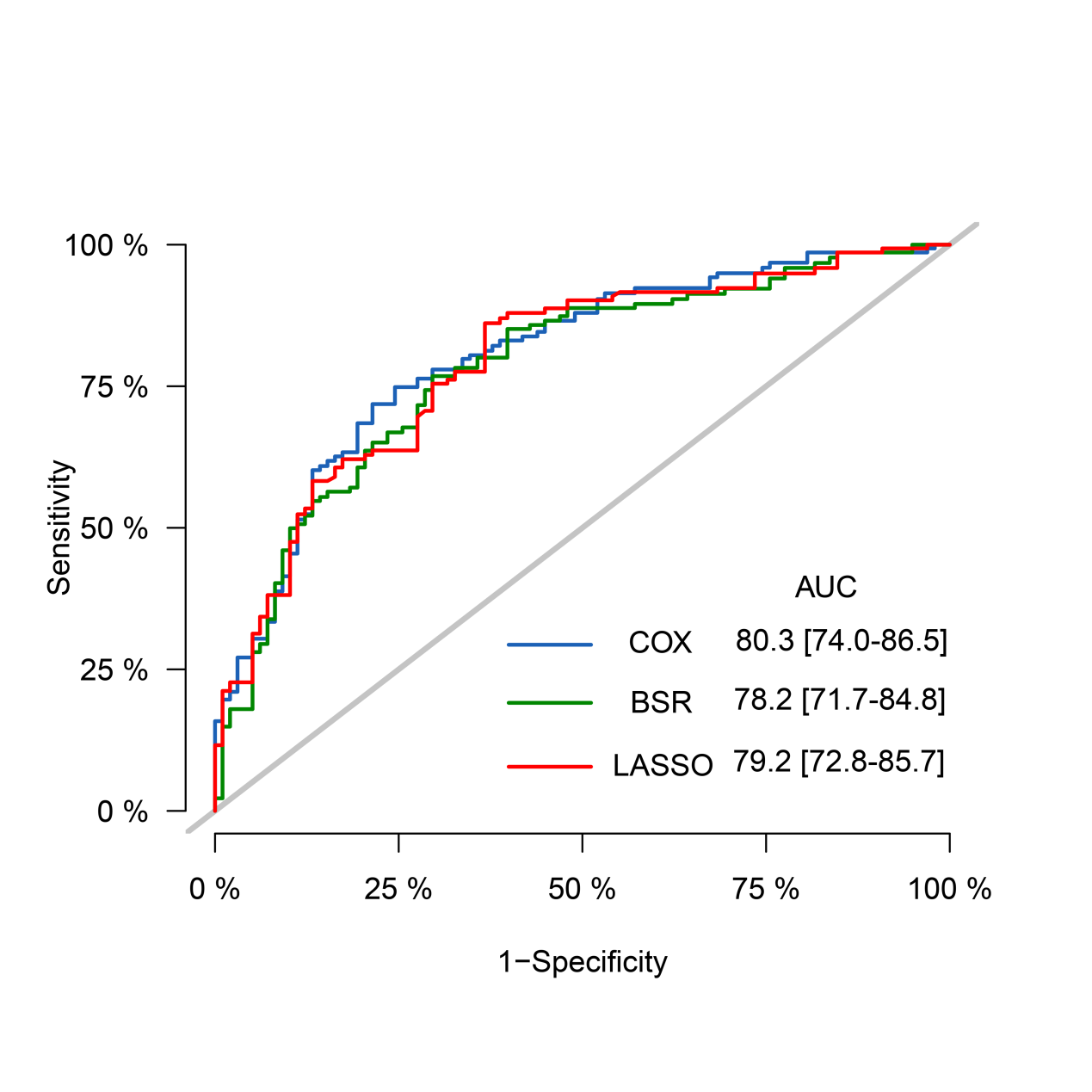

Supplement: oyad107_suppl_Supplementary_Material [file oyad107_suppl_supplementary_material.docx]
